# The Importance of Sphingosine Kinase in Breast Cancer: A Potential for Breast Cancer Management

**DOI:** 10.7759/cureus.13413

**Published:** 2021-02-18

**Authors:** Dutt S Patel, Farrukh Ahmad, Majdi Abu Sneineh, Ravi S Patel, Sai Rohit Reddy, Adiona Llukmani, Ayat Hashim, Domonick K Gordon

**Affiliations:** 1 Internal Medicine, California Institute of Behavioral Neurosciences & Psychology, Fairfield, USA; 2 Emergency Medicine, California Institute of Behavioral Neurosciences & Psychology, Fairfield, USA; 3 Emergency Department, Beaumont Hospital, Dublin, IRL; 4 Internal Medicine, Scarborough General Hospital, Scarborough, CAN

**Keywords:** sphingosine kinase, sphingosine-1-phosphate, breast neoplasms

## Abstract

Breast cancer management includes a combination of surgery, radiation therapy, and chemotherapy. While this management has proven effective, it is not perfect. To expand the umbrella of management to resistant breast cancer tumors, researchers have explored the idea of sphingosine kinase (SphK) and sphingosine-1-phosphate (S1P) as a potential target for treatment. In this article, we review the mechanism of the sphingosine kinase/sphingosine-1-phosphate (SphK/S1P) axis along with its effect on the tumor microenvironment (TME) and compounds that have been studied inhibiting the SphK/S1P axis. We searched for relevant articles in the last five years in Medline and PubMed Central. Inclusion criteria, exclusion criteria, and quality checklists were applied to identify the most relevant articles. We compiled the information that has been summarized in the respective tables and figures provided in this review. The metabolism of sphingolipids was summarized, followed by the SphK/S1P upregulation in breast cancer cells. The variety of effects by upregulation of SphK led to an increase in inflammation, growth, and metastasis in breast cancer tumors. The increase in S1P also impacted the TME, including the cells and surrounding tissue, allowing the breast tumors to thrive. The final point made was a summary of the compounds and drugs that inhibited the SphK/S1P axis. They have proven their effectiveness and show even greater efficacy in combination with docetaxel and doxorubicin in preclinical studies. In conclusion, what is known about the SphK/S1P axis within breast cancer cells is immense but incomplete as we summarize what is known so far. Having a complete picture will allow a faster transition to application in the clinical field but clinical trials have not commenced as of yet.

## Introduction and background

Breast cancer is the second most diagnosed cancer in women living in the United States after skin cancer [1]. It is estimated that in 2020, 30% of all cancers in women will be diagnosed as breast cancer, according to the National Breast Cancer Foundation [2]. While breast cancer mortality has decreased in the last few decades due to advances in detection and management, it is still the second leading cause of cancer death in women [3]. Decreased mortality due to better management can be linked to the identification of molecular markers, gene profiles, and targeting specific receptors. Most breast cancers are estrogen receptor (ER) and progesterone receptor (PR) positive (70%), while some overexpress human epidermal receptor 2 (HER2). Approximately 15-20% of breast cancers are negative for all three receptors, known as triple-negative breast cancer (TNBC). This type has the worst prognosis due to aggressiveness or metastasis and lack of targeted treatment [4]. It does not respond to hormonal or antibody therapies like tamoxifen or trastuzumab, making chemotherapy the primary choice for management. Currently, such tumors are treated with a combination of surgery, radiation therapy, and chemotherapy. While this therapy can improve survival rates, many side effects reduce quality of life and even interfere with further cancer treatment [4].

To manage resistant breast cancer more efficiently, researchers have tried to tackle this issue from different angles. One angle researched over the past two decades is the role of lipid phosphates in the microenvironment of cancerous tumors [5]. Of the lipid phosphates and their corresponding lipid phosphate phosphatases (LPP), the primary focus is on how sphingosine-1-phosphate (S1P) and sphingosine kinase (SphK) upregulation can promote the growth of a tumor via angiogenesis and lymphangiogenesis [6]. By phosphorylation of sphingosine by either isoenzyme, sphingosine kinase 1 (SphK1), or sphingosine kinase 2 (SphK2), S1P is formed [5]. S1P generated by SphK1 impacts cancer progression because the S1P formed is exported out of the cell to act on its specific cell surface G-protein-coupled receptors (S1P receptors 1-5). Of the receptors, S1P receptor 1 (S1PR1), S1P receptor 3 (S1PR3), and S1P receptor 4 (S1PR4) are linked with poor prognosis [6]. The S1P acts on these receptors in an autocrine and paracrine fashion in a process called inside-out signaling [5]. The inside-out signaling causes other downstream signals to activate, which increases the growth of new blood vessels and lymphatics. This process has many names, including SphK/S1P, SphK/S1P/S1P receptor, or just S1P axis [4,7]. Without this axis, the tumor would not grow more than 3 mm; all the more reason it is a primary focus for future management [6]. This specific mechanism has become a promising target for future treatment of breast cancer. Currently, novel drugs are being tested with preclinical breast cancer models that target the SphK/S1P axis [3].

While there has been growing research in this area, this mechanism’s applicability for treatment is infantile. It is a relatively novel mechanism, and clarification is needed regarding its potential role in breast cancer management. We hope to shed some light as we review the SphK/S1P axis, focus on the underlying mechanism, and discuss recent developments.

## Review

Methods

Search Strategy and Study Selection

**Figure 1:**
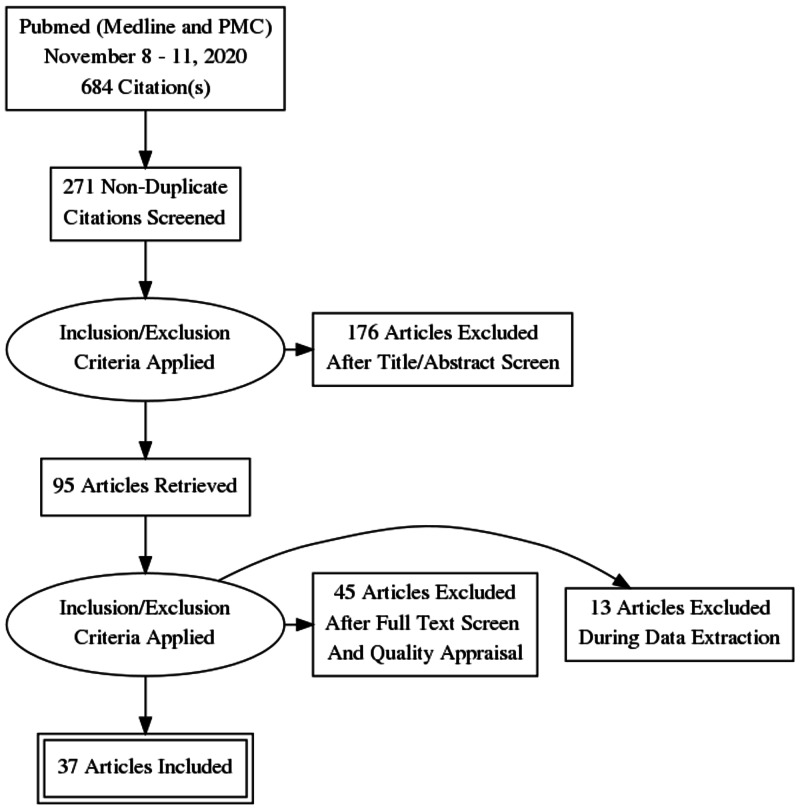
PRISMA flow diagram showing the data selection process. PRISMA: Preferred Reporting Items for Systematic Reviews and Meta-Analyses

PubMed was used to search for relevant publications. The search included the electronic databases Medline and PubMed Central. The keywords used in the search process included “breast cancer and sphingosine kinase,” “breast neoplasm and sphingosine-1-phosphate,” “breast cancer and sphingosine-1-phosphate,” “(breast neoplasm and sphingosine kinase,” “Breast Neoplasms”[Mesh]) AND “sphingosine kinase” [Supplementary Concept], and (“Breast Neoplasms/chemistry”[Mesh] OR “Breast Neoplasms/enzymology”[Mesh]) AND “sphingosine kinase” [Supplementary Concept]. Articles were searched from January 2015 to November 2020 and were imported into Mendeley for organization and citations. After removing the duplicates, all titles and abstracts were reviewed using the inclusion and exclusion criteria. Subsequently, the full-text articles were reviewed, and quality assessment tools were applied for final selection. Figure 1 shows a summary of the study selection process in a Preferred Reporting Items for Systematic Reviews and Meta-Analyses flow diagram.

Study Selection Criteria

The inclusion criteria consisted of: (1) All publications in the last five years (January 2015 to November 2020); (2) articles in only the English language; (3) studies originating from any country; (4) full-text articles; and (5) all studies emphasizing breast cancer and SphK/S1P.

The exclusion criteria consisted of: (1) All publications older than five years (published before January 2015); (2) articles not in the English language; (3) articles not relevant to breast cancer or SphK/S1P; (4) articles not available in full text; and (5) Gray literature.

Results

Search Results and Characteristics

Following the inclusion criteria, 37 studies were used for the review. The studies included 6,627 patient samples, multiple breast cancer cell lines, cats, and mice. Of the 37 included studies, there were 25 cross-sectional studies, eight literature reviews, and four cohort studies. The quality of the studies was checked using the respective quality assessment tool and are shown in Table 1 and Table 2. The cross-sectional studies were assessed using the Newcastle-Ottawa Quality Assessment Scale adapted for cross-sectional studies, and the minimum number of stars to qualify as “high quality” was seven. The Scale for the Quality Assessment of Narrative Review Articles or the SANRA checklist was used to assess the literature reviews. The total score available was 12 and the minimum to qualify as “high quality” was seven. The remaining cohort studies were reviewed using the Newcastle-Ottawa Quality Assessment Scale-Cohort Studies, and the minimum to qualify as “high quality” was seven.

Pathophysiology of Sphingosine Kinase and Breast Cancer

**Figure 2:**
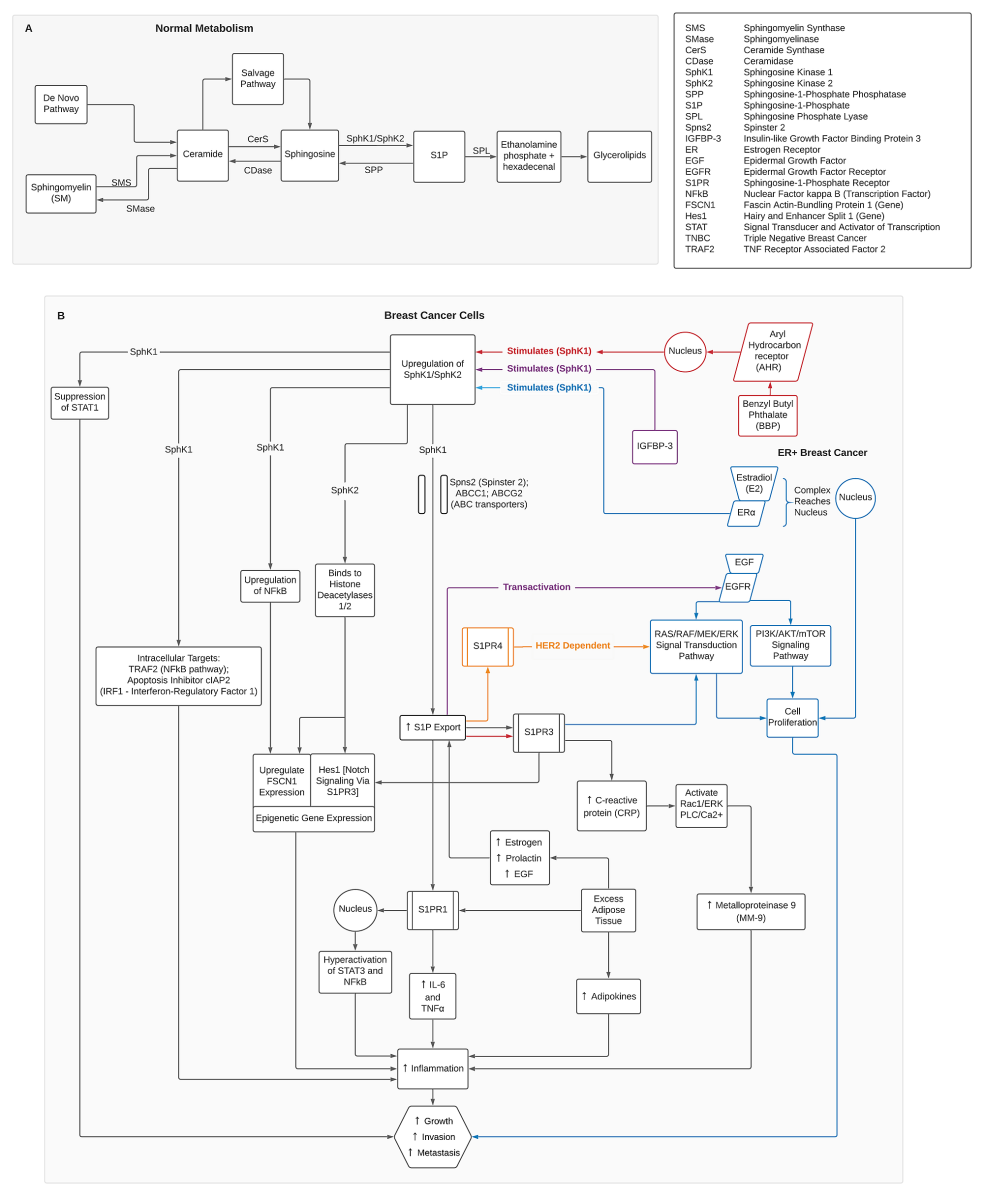
(A) Normal metabolism of sphingolipids. (B) Upregulation of SphK/S1P axis in breast cancer cells.

Information was taken from 27 of the 37 included articles and compiled into a flow chart (Figure 2). A summary of what is known about the normal metabolism of sphingolipids is shown in the upper left portion of the figure. Below the first part of the figure a second flow chart summarizes the pathophysiology of breast cancer cells, emphasizing the role of SphK/S1P in the growth, invasion, and metastasis of cancerous tumors. The figure will be discussed in further detail in the next section. The studies from Table 1 were used to construct Figure 2 and will be discussed accordingly.

Role of Tumor Microenvironment and Promising Compounds

**Figure 3:**
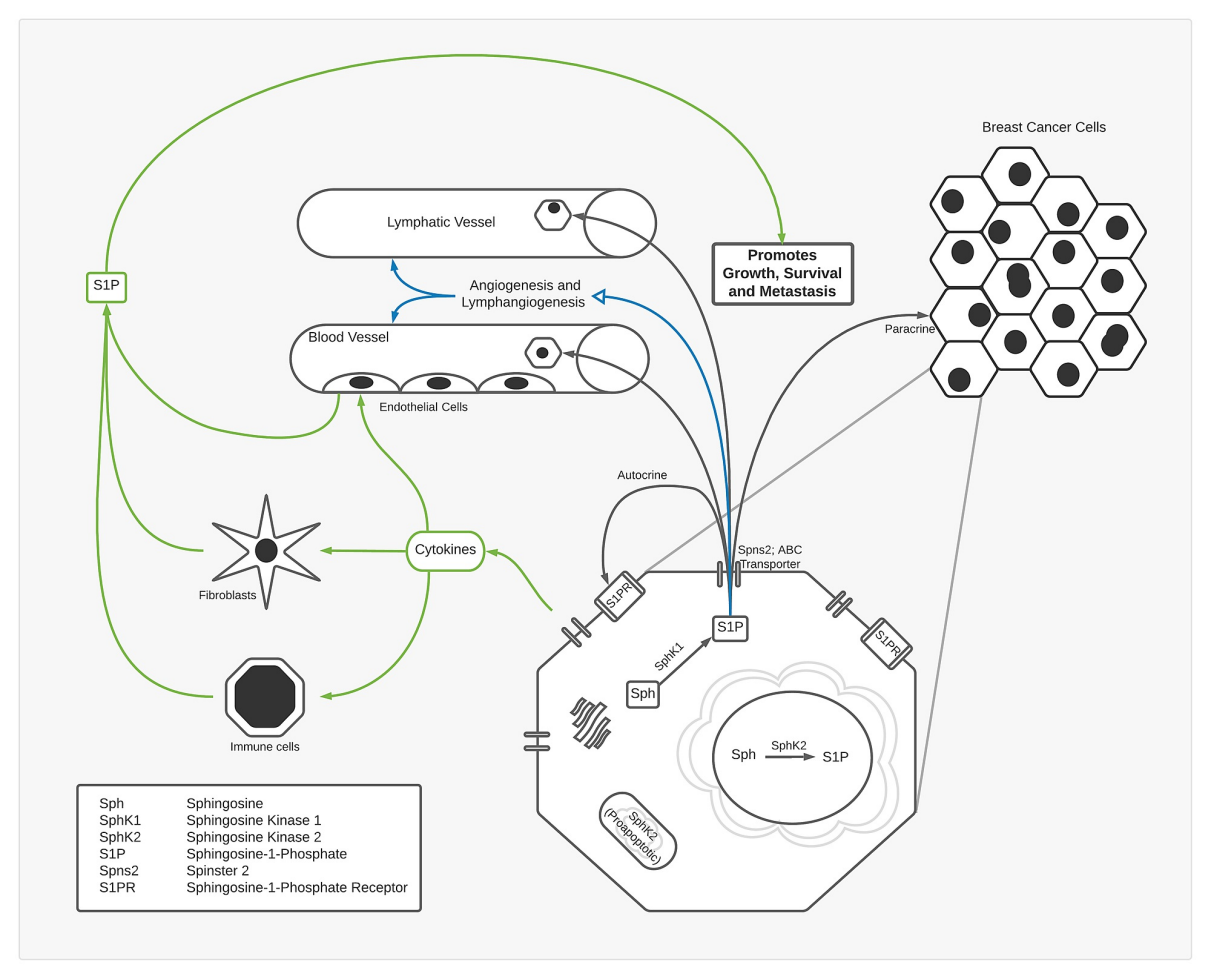
Tumor microenvironment: the effect of the upregulation of SphK on the surrounding tumor cells, vessels, and stroma.

Information was taken from 10 of the 37 included articles to create Figure 3 and Table 1, outlining the significant consequences breast cancer cells have on the tumor microenvironment (TME). The findings will be discussed in the next section. Information from 16 of the 37 articles has been arranged in Table 2, summarizing the compounds or drugs that inhibit the SphK/S1P axis as well as their relevant findings. The details will be discussed in the next section.

 

**Table 1:** Quality assessment of the articles pertaining to pathophysiology and tumor microenvironment. NOS-CS: Newcastle-Ottawa Quality Assessment Scale Cross-Sectional Studies; SANRA: Scale for the Assessment of Narrative Review Articles; NOS-C: Newcastle-Ottawa Quality Assessment Scale Cohort Studies; EMT: epithelial-mesenchymal transition; IL-22R1: interleukin 22 receptor 1; S1PRs: sphingosine-1-phosphate receptors; S1PR1: sphingosine-1-phosphate receptor 1; S1PR2: sphingosine-1-phosphate receptor 2; S1P: sphingosine-1-phosphate; MSC: mesenchymal stem cell; MCP-1: monocyte chemoattractant protein 1; TNBC: triple-negative breast cancer; SphK1: sphingosine kinase 1; SphK2: sphingosine kinase 2; FSCN: fascin actin bundling protein 1; NFkB: nuclear factor kappa B; EDV: endothelium-dependent vessel; VM: vasculogenic mimicry; VE-Cadherin: vascular endothelial cadherin; SGPL1: sphingosine-1-phosphate lyase 1; BBP: benzyl butyl phthalate; AHR: aryl hydrocarbon receptor

First author	Year of publication	Reference	Quality assessment tool	Quality assessment score	Findings/Results
Chen, Z	2020	[8]	NOS-CS	7	SphK1 regulated stem cell characteristics as well as migration, invasion, and induced EMT in breast cancer cells
Kim, EY	2020	[9]	NOS-CS	7	In highly metastatic breast cancer cells, there was an increased expression of both IL-22R1 and S1PR1. IL-22 enhanced the expression in the receptors of breast cancer cells while also increasing S1P and MCP-1 in MSCs
Oshi, M	2020	[10]	NOS-C	7	The Tumor Angiogenesis Score was evaluated, and a high score was associated with increased expression of S1P-related genes, hypoxia, increased angiogenesis, increased expression of inflammation-related genes, metastatic redundancy, and decreased infiltration of immune cells. A high score was not associated with overall survival, aggressive features, or response to neoadjuvant chemotherapy
Sakharkar, MK	2020	[11]	NOS-CS	7	Evaluation of 36 genes related to sphingolipid metabolism was completed in both breast cancer cells and healthy controls. Five genes were upregulated while the *S1PR1* gene was downregulated in breast cancer cells. The gene pair correlation coefficient was high in the control group but lost their correlation in breast cancer patients. No genes were identified as having a strong prognostic value
Singh, SK	2020	[4]	SANRA	9	Summarized current therapy available forTNBC followed by S1P metabolism and the potential for S1P to be a target for future therapy. Importance of S1P in chemotherapy-induced peripheral neuropathic pain and cancer-related bone pain was emphasized
Zhong, L	2020	[12]	NOS-CS	7	Lower S1PR1 expression correlated with a poor overall survival in breast and lung carcinomas, while increased S1PR1 expression correlated positively with immune cell infiltration
Acharya, S	2019	[13]	NOS-CS	7	TNBCs had a high expression of SphK1 and promoted lung metastasis while blocking expression produced the opposite effect. FSCN1, a contributor to metastasis, was found to be upregulated by SphK1 through the activation of NFkB. By inhibiting the pathway, tumor growth and lung metastasis were inhibited as well
Liu, S	2019	[14]	NOS-CS	7	Upregulation of S1PR1 increased EDV while inhibiting VM by increasing β-catenin expression leading to decreased VE-cadherin. S1PR1 also increased VE-cadherin phosphorylation promoting the separation of VE-cadherin from β-catenin
Alshaker, H	2019	[15]	NOS-CS	7	RNA transcriptome microarray technology was used to understand the effects of SphK knockdown. RAS, MAPK, GTPase, Wnt, and PI3K were some of the pathways which were upregulated in the absence of SphK1 and SphK2, while other pathways involved in cancer were unaffected
El Buri, A	2018	[16]	NOS-CS	7	Breast cancer stem cell lines released S1PR2 in which the exosomal S1PR2 activated fibroblasts.The processing of exosomal S1PR2 by fibroblasts was also identified
Engel, N	2018	[17]	NOS-CS	7	SGPL1 was identified in the cytoplasmic membrane. SGPL1 levels were low in breast cancer cells. High SGPL1 levels prevented S1P stimulation
Shimizu, Y	2018	[18]	NOS-CS	7	Lack of SphK1 causes a decrease in expression of claudin-2 in HER2/neu-induced breast tumors as well as reduction in overall tumor development. Increased SphK1 expression promoted cancerous cellular functions in the same breast cancer cells
Wang, S	2018	[19]	NOS-CS	9	Hyperexpression of SphK1 was seen in breast cancer tissues and correlated with high levels of S1P. Inhibiting SphK1 decreased metastatic potential in breast cancer cell lines. Increased S1P levels was identified to activate Notch signalling through the S1PR3
Yamada, A	2018	[20]	NOS-CS	7	Increased expression of ABCC1 transporter increased S1P secretion, metastatic potential, enhanced tumor growth, and the S1P exported by the transporter increased transcription of SphK1. This contributed to a poor prognosis in breast cancer patients. On the contrary, ABCB1 was not associated with any of these effects
Do, SI	2017	[21]	NOS-C	7	SphK1 was proven to have overexpression in breast cancer tissue compared to normal mammary tissue showing association with higher metastatic potential and aggressiveness
Calis, IU	2017	[22]	NOS-CS	7	Lateral motility, adhesion and proliferation, and viability of breast cancer cell lines were statistically reduced by silencing S1PR1 and S1PR3 genes. The silencing of both genes was greater than silencing either gene individually
Maia, LP	2017	[23]	NOS-CS	7	S1PRs in breast cancer patients and tumors were expressed in lymphocytes, monocytes, and granulocytes at varying levels. Variable expression of genes of sphingolipid metabolism and cytokine production was also identified
Nazouri, AS	2017	[24]	NOS-CS	7	High SphK1 expression was associated with a higher BMI in breast cancer tumors, including tumors without receptor expression
Zhu, YJ	2017	[25]	NOS-C	8	Correlation between increased SphK1 mRNA expression and increased breast cancer proliferation, metastasis, EGFR2, and shorter overall survival was statistically proven
Ko, P	2016	[26]	NOS-CS	7	SphK1 expression was shown to depend on extracellular matrix rigidity in breast cancer cell lines present in conditioned media. Reducing lipid components increased the invasive activity of these breast cancer cells, but silencing SphK1 did not inhibit invasive capacity in the conditioned media
Wang, YC	2016	[27]	NOS-CS	7	BBP acted on the AHR that used SphK1 signaling to promote breast cancer cell growth and metastasis to the lungs. S1PR3 was identified to stimulate the growth of the breast cancer cells

**Table 2:** Compounds/drugs inhibiting the SphK/S1P axis tested in the articles reviewed. NOS-CS: Newcastle-Ottawa Quality Assessment Scale Cross-Sectional Studies; SANRA: Scale for the Assessment of Narrative Review Articles; NOS-C: Newcastle-Ottawa Quality Assessment Scale Cohort Studies; CSO: coix seed oil; FTY720: fingolimod; TNBC: triple-negative breast cancer; S1PR1: sphingosine-1-phosphate receptor 1; SKI-I: sphingosine kinase inhibitor 1; SKI-II: sphingosine kinase inhibitor 2; SK1-I: sphingosine kinase 1 inhibitor-I; S1PR4: sphingosine-1-phosphate receptor 4; DTX: docetaxel; SphK1: sphingosine kinase 1; S1P: sphingosine-1-phosphate; HDACs: histone deacetylases; ER: estrogen receptor; CNP: complex nanoparticle; S1PRs: sphingosine-1-phosphate receptors; SphK2: sphingosine kinase 2; S1PR2: sphingosine-1-phosphate receptor 2

First author	Year of publication	reference	Quality assessment tool	Quality assessment score	Drugs/Compounds mentioned	Findings/Results
Hii, LW	2020	[28]	NOS-CS	8	FTY720	FTY720 and PF543 both increased doxorubicin sensitivity in breast cancer cell lines
					PF543	
Fang, T	2020	[29]	NOS-CS	8	CSO	CSO downregulated the expression of S1PR1, cyclinD1, and phosphorylated levels of STAT3, MAPK, and AKT while upregulating p27. This led to decreased growth and viability of breast cancer cells
Rupp, T	2020	[30]	NOS-CS	7	FTY720	FTY720 suppressed growth and viability of TNBC in both in vitro and in vivo models
Alshaker, H	2020	[31]	SANRA	10	SKI-I	SKI-I inhibited growth without a high degree of side effects
					SKI-II	SKI-II modified the HER2 pathway and reduced ERK1/2 activation via S1PR4. Likewise, it increased sphingosine within the cell and reduced growth while increasing apoptosis
					SK1-I	SK1-I decreased angiogenesis and lymphangiogenesis while decreasing tumor size
					PF543	PF543 reduced metastasis and invasiveness of the tumor
					SK1-5C	SK1-5C reduced ERK1/2 and AKT pathways while decreasing growth and size and promoting apoptosis
					SK-F	SK-F reduced cellular growth sensitizing breast tumors in mice to DTX while showing no added adverse effects
					FTY720	FTY720 alone showed limited efficiency, but with doxorubicin or lower doses of DTX, higher efficacy ensued
Nagahashi, M	2018	[32]	NOS-CS	7	FTY720	FTY720 inhibited SphK1/S1P/S1PR1 axis decreasing inflammation, breast cancer proliferation, and lung metastasis caused by obesity
Alshaker, H	2018	[33]	NOS-CS	7	Controls: SKI-178, 12aa, and SK1-I	SKI-178, 12aa, and SK1-I are known SK1 inhibitors used to find six new compounds with similar effects, SK-A, SK-B, SK-C, SK-D, SK-E, SK-F (field template analysis); SK-C, SK-E, SK-F inhibited SK1 in a cell model, with SK-F having the highest inhibition percentage
					6 new: SK-A to SK-F	There was no significant change in tumor size or viability with the use of SK-F alone compared to the control group, but there was a significant reduction with DTX
Geffken, K	2018	[3]	SANRA	10	FTY720	Phosphorylated FTY720 inhibits HDACs allowing ER to form in ER-negative breast cancer. Unphosphorylated FTY720 inhibits SphK1 by directly binding to the enzyme and therefore blocks all of S1P’s effects
Alshaker, H	2017	[34]	NOS-CS	8	FTY720-docetaxel nanoparticle	FTY720 resensitized breast cancer cells to DTX reducing the effective dose by four. CNP had the same effect as the control but lowered the dose needed and reversed the side effects of weight loss, liver toxicity, and lymphopenia
Katsuta, E	2017	[35]	NOS-CS	7	FTY720	After treatment with doxorubicin, patient samples showed an upregulation of STAT3 which was suppressed when doxorubicin was given with FTY720. Doxorubicin-resistant breast cancer cell lines showed a high expression of SphK1 which was inhibited with the help of FTY720 causing a decrease in growth
Ochnik, AM	2017	[36]	NOS-C	8	SKI-II	Individually both OSI-906 and SKI-II produced positive results in a dose-dependent manner. Synergism was confirmed by reducing cell viability better than each individual drug
Hait, N C	2017	[37]	SANRA	9	SKI-I	SKI-I, K-145, PF-543, and DMS exerted some beneficial effects on breast cancer. FTY720 has shown that the phosphorylated form accumulating in the nucleus inhibits class I HDACs and can cause ER to form in ER-negative breast cancer
					K-145	
					PF-543	
					DMS	
					FTY720	
Tsuchida, J	2017	[38]	SANRA	9	FTY720	FTY720 reduced lymphocyte trafficking by binding of the phosphorylated form to lymphocytes and endothelial cells.
					BML-258	BML-258 decreased growth, size, and metastasis to the lung
					Sonepcizumab	Sonepcizumab, a monoclonal antibody against S1P, reduced size and proliferation, while a few cases showed complete disappearance of the tumor
Nakajima, M	2017	[5]	SANRA	9	FTY720	FTY720 reduces expression of S1PR1 while also reducing overall levels of S1P
					SK1-I	SK1-I decreased angiogenesis and lymphangiogenesis
Hait, NC	2015	[39]	NOS-CS	7	FTY720	FTY720 is phosphorylated by SphK2 in the nucleus causing an inhibition of histone deacetylases. This leads to suppression of tumor growth and development, while also sensitizing tamoxifen to ER-negative breast cancer
Mukhopadhyay, P	2015	[6]	SANRA	7	SK1-I	SK1-I inhibits angiogenesis and lymphangiogenesis
					PF543	PF543 is not effective without the presence of TME
					FTY720	FTY720 binds to all S1PRs after phosphorylation by SphK2 except S1PR2. Likewise, it accumulates in the nucleus and may regulate gene expression of ER
Marzec, KA	2015	[40]	SANRA	8	SKI-II	SKI-II inhibits SphK1, and in combination with gefitinib (EGFR inhibitor), produced a synergistic effect

Discussion

The SphK/S1P axis is a relatively new concept that has the potential to be a factor in the future of breast cancer management. While there are currently modes of treatment available for breast cancer, it is still insufficient in treating complicated and severe cases. The information about the SphK/S1P axis has been summarized below. As new research is published every year, we discuss what is known so far and what information is still needed for future integration into breast cancer management. It is important to note that all studies mentioned were preclinical.

Metabolism of Sphingolipids

The top part of Figure 2A summarizes the metabolism of sphingolipids in the absence of cancer. Ceramide is a paramount sphingolipid molecule and is formed through many processes [4]. These include de novo synthesis, formation from sphingomyelin, and a particular product in the salvage pathway [37]. The de novo synthesis can occur in the endoplasmic reticulum, lysosomes, and mitochondria, where a series of reactions occur to produce ceramide. Ceramide can also form from sphingomyelin with the help of sphingomyelinase. Likewise, ceramide can form right before the salvage pathway begins. Glucosylceramide, which is formed from ceramide for the salvage pathway by glucosylceramide synthase, can be reversed by glucocerebrosidase. Ceramide is then converted to sphingosine by ceramidase. Sphingosine is also formed indirectly from ceramide via the salvage pathway.

S1P is finally formed from sphingosine by SphK. SphK1 and SphK2 are the two essential isoforms present in different organelles of the cell. SphK2 is present within the nucleus and the mitochondria, while SphK1 is present within the cytosol. S1P is then broken down by sphingosine phosphate lyase (SPL) to form ethanolamine phosphate and hexadecenal, further breaking down into glycerolipids [4]. Some enzymes reverse the process to balance the substrate and product level, keeping the cell environment in homeostasis [37]. These enzymes include sphingomyelin synthase, which converts ceramide back to sphingomyelin, and ceramide synthase (CerS), which converts sphingosine to ceramide. Likewise, sphingosine-1-phosphate phosphatase (SPP) converts S1P back to sphingosine [37].

SphK/S1P Axis in Breast Cancer

SphK, in a typical cell environment, functions in homeostasis. However, in breast cancer cells, studies have shown an upregulation of SphK1/SphK2 [31]. Figure 2B summarizes what is known about the mechanism behind this cascade. The overall outcome is displayed at the bottom of the figure conveying an increase in inflammation, growth, invasion, and breast cancer metastasis.

The upregulation of SphK1 has been thoroughly linked with poor prognosis in breast cancer [8,19,21,24,25]. SphK1 upregulation causes an increase of S1P in the cytosol. Likewise, the levels of S1P remain high due to the downregulation of SPL. A recent study found breast cancer cells to have low levels of an SPL variant in the cytosol, allowing S1P to stay active [16]. After formation, S1P is excessively exported with the help of specifically identified transporters to the TME. These upregulated transporters include Spinster 2 (Spns2) and specific ATP-binding cassette transporters (ABC transporters), including ABCC1, ABCG2, and ABCC11 [38]. Not all ABC transporters are involved, for example, ABCB1, which does not correlate with increased S1P secretion, breast cancer tumor growth, or angiogenesis [20].

The role of S1P is the cornerstone of breast cancer proliferation. The hyperregulation of S1P has been linked to inflammation, cancer cell survival, tumor growth, metastasis, angiogenesis, lymphangiogenesis, chemokine signaling, epigenetic regulation, and immune cell trafficking [37]. Moreover, a 2020 study determined the increase in S1P due to the upregulation of SphK1 caused an increase in epithelial-mesenchymal transition (EMT) [8]. EMT is the loss of differentiation and intercellular junctions, causing the cells to have mesenchymal cell characteristics while being more prone to metastasis.

Once outside the cell, S1P works via autocrine and paracrine action on the S1PRs. As described earlier, there are five subtypes of S1PRs, but not all have been linked to breast cancer promotion. S1PR1, S1PR3, and S1PR4 have been linked to cancer proliferation thus far [3]. Extracellular S1P exerts its inflammatory effects via S1PR1 and S1PR3. The S1P effect on S1PR1 causes an increase in IL-6 and TNFα and increases the activation of signal transducer and activator of transcription 3 (STAT3) and nuclear factor-kappa B (NFkB) within the nucleus, all of which contribute to the inflammation [6,23]. Through the effect of S1PR1, obesity can lead to an increase in inflammation and breast cancer proliferation as well [24,32]. The excess adipose tissue increases estrogen, prolactin, and epidermal growth factor (EGF), creating a downstream effect by increasing S1P.

Similarly, there is an increase in adipokines in correlation with obesity, contributing to breast cancer growth. Furthermore, S1P binding to S1PR3 increases inflammation by increasing C-reactive protein (CRP). The increased CRP leads to activation of signaling pathways Rac1/ERK and PLC/Ca2+, thereby causing an upregulation in metalloproteinase 9 (MM-9) expression. Increased MM-9 expression has been linked to increased inflammation as well as increased invasion and metastasis [31]. A study reinforced the importance of S1PR1 and S1PR3 in breast cancer growth and adhesion by silencing the genes for these two receptors, resulting in a statistically reduced growth in the breast cancer tumors [22].

Estrogen receptor (ER) status is another crucial factor regarding the SphK/S1P axis. In ER-positive breast cancer, estradiol (E2) binds to ERα, which upregulates SphK1, causing an increase in S1P. Once exported, S1P acts on S1PR3, which stimulates ERK1/2 of the RAS/RAF/MEK/ERK signal transduction pathway leading to cancer cell proliferation [31]. S1P and S1PR3 may also stimulate AKT of the PI3K/AKT/mTOR signaling pathway producing a similar effect [15]. Simultaneously, the E2-ERα complex reaches the nucleus increasing certain transcription factors, and, in turn, promotes breast cancer cell growth. Another study found that high levels of S1PR1 and S1PR3 in ER-positive breast cancer cells resulted in a poor prognosis [22]. This evidence reinforces the importance of ER status in correlation to the SphK/S1P axis.

For ER-negative breast cancer cells, evidence shows a human EGF receptor 2 (HER2)-dependent pathway causes S1PR4 to stimulate the ERK signal pathway. An additional study proved that by downregulating SphK1, the growth of HER2 breast tumors reduced as well. The tumor reduction also correlated with a decrease in claudin-2 expression and may have been an underlying factor [18]. On the other hand, there has also been evidence that S1P levels were lower in the presence of HER2 upregulation [37,38]. The mixture of evidence indicates a need for further research to determine the correlation between HER2 and the SphK/S1P axis.

Concerning EGFR downstream signaling pathways, evidence shows that insulin-like growth factor binding protein-3 (IFGBP-3) upregulates SphK1 [40]. IFGBP-3 essentially uses the same mechanism previously stated (exported S1P acting on the respective receptor) to induce the same signal transduction pathways. The result is an increase in the growth of breast cancer cells and a worse prognosis. This mechanism needs more clarity, though, as one study revealed mixed results. While there was a correlation between gene expression of insulin-like growth factor receptor (IGFR) and SphK1, the protein expression of IGFR and SphK1 did not correlate [36].

S1P not only exerts its effects extracellularly via S1PRs but also intracellularly through different mechanisms. In a recent study, cytosolic S1P generated from SphK1 suppressed signal transducer and activator of transcription 1 (STAT1)-mediated interferon (IFN) signaling to allow excessive growth. STAT1 has shown tumor-suppressing properties, and when SphK1 was decreased, STAT1 signaling increased as well, leading to cell apoptosis [28]. Cytosolic S1P also has intracellular targets that induce inflammation. The targets include a component of the NFkB pathway, TNF receptor-associated factor 2 (TRAF2), as well as an integrant of the interferon regulatory factor 1 (IRF-1) mediator of inflammation, apoptosis inhibitor cIAP2 [37]. SphK1 regulates other elements as well. Fascin actin-bundling protein 1 (FSCN1) is a gene that promotes metastasis and was shown to upregulate by SphK1 via NFkB in a recent study [13]. Another gene that is upregulated by SphK1 is hairy and enhancer split 1. It is a component of the Notch signaling pathway whose regulation is mediated through S1PR3 [19,31]. Other genes upregulated by the SphK1/S1P axis in breast cancer have been identified, including CERS1, CERS2, CERS6, and UGCG [11]. Likewise, this epigenetic regulation can also be regulated by SphK2. SphK2 is present in the nucleus where the S1P formed binds to histone deacetylases 1 and 2, essentially inhibiting them. In turn, this epigenetic regulation causes breast cancer progression via histone acetylation and gene transcription [37]. It is important to mention that while upregulation of SphK2 in the nucleus may cause cancer to progress, SphK2 in the mitochondria has the opposite effect. S1P formed in the mitochondria is degraded by SPL to hexadecenal. The product then binds to an apoptosis regulator, promoting the release of cytochrome c [37]. Increased cytochrome c leads to apoptosis and inhibits cancer growth.

Other factors have been discovered to cause an upregulation of SphK1, leading to breast cancer progression. One study identified benzyl butyl phthalate (BBP) promoting breast cancer cell growth by the same mechanism. In a dose-dependent manner, BBP binds to the aryl hydrocarbon receptor (AHR) and stimulates transcription factors, causing an increase in S1P produced by SphK1. S1P subsequently binds to S1PR3 to cause breast cancer growth [27]. A different study decreased lipid concentration in the extracellular matrix to increase its rigidity, resulting in an upregulation of SphK1 [26]. This result increased the metastatic potential and proved to be another factor manipulating the SphK/S1P axis.

SphK1/SphK2 hyperregulation has many downstream consequences that have been identified and replicated. This discussion only took into account what has been published in the last five years. Figure 2 does not cover every detail of the SphK/S1P axis in breast cancer cells, as this concept is intricate. In this section, the intracellular and extracellular ramifications of the SphK/S1P axis were discussed, but the next section considers the larger environment in which the breast tumor thrives.

Tumor Microenvironment

While understanding the upregulation of the SphK/S1P axis and its sequelae is essential, what is paramount is the environment in which breast cancer cells proliferate. The main components of the TME include blood vessels, lymphatics, and stromal cells. Figure 3 shows a basic summary of the effects of SphK/S1P axis on the TME and how these effects lead to metastasis, angiogenesis, lymphangiogenesis, and immune cell trafficking [37]. Before summarizing the effects of S1P on the TME, appreciating the quotidian environment is vital. S1P levels remain in homeostasis due to the balance between SphK and SPP as well as SPL. While overexpression of SphK can lead to overgrowth and cancer, SPP overexpression can lead to apoptosis [5]. In the absence of cancer, S1P levels in the blood are maintained mainly by erythrocytes and complemented by vascular endothelial cells, according to recent evidence [5]. Endothelial cells of the lymphatics also regulate S1P levels with the help of the Spns2 transporter. Likewise, stromal and immune cells are likely to be a source of production in homeostasis, but more evidence is required.

In the presence of breast cancer cells, S1P levels increase in the TME, causing an imbalance and leading to a cascade of effects. Figure 3 (bottom) represents a breast cancer cell exporting S1P into the TME. The first consequence has been described in detail previously as S1P induces growth and proliferation of the tumor cells and metastasis in an autocrine and paracrine manner via transporters and S1PRs.

The second significant repercussion is on the promotion of angiogenesis and lymphangiogenesis. One study indicated that S1PR1 was responsible for angiogenesis by activating vascular endothelial growth factor receptor, EGFR, and platelet-derived growth factor receptor [3]. It can also be concluded that a vicious cycle can occur because these growth factors can upregulate SphK1. An additional study in 2020 crreated an angiogenesis pathway score utilizing genes associated with S1P to look for a correlation with metastasis, aggressiveness, and inflammation [10]. On the other hand, a recent article studied tumor angiogenesis by contrasting endothelium-dependent vessel (EDV) and vasculogenic mimicry (VM) per S1PR1, the latter associated with a worse prognosis. The study found that by depleting S1PR1, EDV was impaired while VM intensified, leading to a worse prognosis in breast cancer [14]. Another study found similar results in which decreased levels of S1PR1 correlated to a poorer prognosis [9]. Regardless of these results, most research points to a positive correlation between S1P, S1PR1, and angiogenesis. Though there is a large proportion of research regarding angiogenesis, the opposite is true for S1P and lymphangiogenesis [3]. There is plenty evidence regarding metastasis via lymphatics in breast cancer, so lymphangiogenesis can be assumed to have a role, but further research is needed to find the underlying mechanisms.

Lastly, S1P released from breast cancer cells can induce S1P production from non-cancer cells in the TME. This induction is accomplished by increasing cytokines and growth factors mentioned previously, along with fibroblast growth factor (FGF). Along with the cytokines mentioned before, a recent study found a correlation between IL-22 receptor and S1PR1 in highly metastatic, advanced breast cancer tumors [12]. The cells affected by these cytokines and FGF can include endothelial cells, fibroblasts, and immune cells. A study found that breast cancer patients had granulocytes, monocytes, and lymphocytes expressing high levels of S1PRs [23]. An additional study showed that S1PR2 from breast cancer stem cells activated fibroblasts [16]. All these cells affected by S1P produce S1P themselves, causing a domino effect increasing survival and growth of the tumor multi-fold.

The combination of effects caused by the SphK1/S1P axis at the cellular level with the surrounding environment is why this mechanism is salient. It has the potential to be a key target for treatment because inhibiting SphK can halt breast cancer progression at each of these levels. The next section discusses the compounds/drugs that inhibit the SphK/S1P axis in one form or another.

Promising Compounds/Drugs

By utilizing the SphK/S1P axis, researchers have tested compounds and drugs that hinder breast cancer tumors. Table 2 summarizes the findings of each study in this review. This aspect is the most novel as no drugs have been used in clinical trials on a large scale. The mentioned studies assess the compounds’ efficacy on breast cancer tissue samples, breast cancer cell lines, and mice.

The compounds have been previously classified into SphK inhibitors that inhibit both SphK1 and SphK2 (Pan-SphK inhibitors) and those that selectively inhibit SphK1 [31]. These pan-SphK inhibitors include sphingosine kinase inhibitors 1 and 2 (SKI-I and SKI-II) and N,N-dimethyl-D-erythro-sphingosine (DMS). Most of the other compounds inhibit only SphK1 and include FTY720, PF543, SKI-178, 12aa, SK1-I, SK-A to SK-F, SK1-5C, and BML-258. K-145 inhibits SphK2 and sonepcizumab is the only monoclonal antibody against S1P.

Many studies tested FTY720 (fingolimod) which was approved by the Food and Drug Admininstration for use in multiple sclerosis previously [3]. FTY720 has been shown to bind to SphK1, blocking S1P formation in the cytosol directly [3]. On reaching the nucleus of a breast cancer cell, it is phosphorylated by SphK2, inhibiting histone deacetylases. This inhibition causes an upregulation of genes concerned with ER, which otherwise have been suppressed in ER-negative breast cancer [3,6,30,37,39]. FTY720 was also shown to inhibit obesity-related inflammation, proliferation, and metastasis by inhibiting S1PR1 [29,40]. One study found that the phosphorylated FTY720 also reduced lymphocyte trafficking by binding to lymphocytes and endothelial cells, essentially affecting the TME [38]. This drug has been shown to inhibit the growth of breast cancer cells but with limited efficacy alone [35]. Studies have shown that in combination with doxorubicin or docetaxel (DTX), the dose needed for each drug was reduced, while the incidence of side effects of weight loss, liver toxicity, and lymphopenia also reduced [28,31,34]. The mechanism of FTY720 is well established and has been proven to reduce breast tumor size and growth in coordination with other modes of treatment for breast cancer. Moving on to more extensive studies with more subjects or even randomized control trials may be the next step.

Other compounds inhibiting SphK1 include PF543, which has been shown to reduce invasiveness and metastasis of breast cancer tumors; however, one study found that PF543 is not efficacious without the presence of the TME [6]. The result indicates the need to test these compounds in vivo amply to see the overall effect. In a separate study, PF453 was proven to improve doxorubicin sensitivity in breast cancer cells, further demonstrating its efficacy [28]. SK1-I is another selective SphK1 inhibitor that has been shown to reduce angiogenesis and lymphangiogenesis [5,6,31]. SK1-I was another compound that inhibited the growth of breast cancer tissue via the TME. SK-F is a recently discovered compound with a similar mechanism, which showed no statistically significant change in tumor proliferation individually, but decreased tumor size in the presence of DTX at lower doses [33]. Further studies in the future may give more insight into this compound. BML-258 has been shown to inhibit SphK1 and decrease growth, size, and metastasis of the breast tumors [38]. SK1-5C has shown similar results by decreasing ERK1/2 and AKT pathways.

Similarly, the pan-SphK inhibitors, SKI-I and SKI-II, and DMS have been beneficial in breast cancer inhibition. SKI-I was shown to reduce the tumor growth without a high degree of side effects [31]. SKI-II was tested in combination with EGFR inhibitor, gefitinib, and a dual IGF1R/insulin receptor tyrosine kinase inhibitor, OSI-906, in two separate studies. Each study resulted in a synergistic effect in a dose-dependent manner [36,40]. K-145 was the only selective SphK2 inhibitor mentioned in the reviewed studies showing beneficial effects [37]. Likewise, the only monoclonal antibody against S1P was sonepcizumab, which also reduced growth and size, while, in some cases, caused a complete disappearance of the breast tumor [38]. One study analyzed coix seed oil (CSO) and showed that it decreased the expression of S1PR1, cyclin D1, phosphorylated MAPK, and AKT while at the same time upregulating p27. The effect of CSO caused a decrease in viability and inhibited the growth of the breast cancer cell and induced apoptosis [29]. Although these compounds have shown value in different aspects, more studies are required to prove whether they can be used to manage breast cancer in the future.

With the research and information discussed, these compounds have potential but they lack clinical evidence. While many studies have investigated some compounds, many still lack collective evidence. Still, these compounds are promising, especially when complimenting other drugs. Where modern treatment stops, this mechanism can eventually have an impact if it reaches clinical trials. Currently, there are no active clinical trials pertaining to any SphK inhibitors and their impact in breast cancer [41].

While we strive for the most up-to-date and accurate information, there are still limitations concerning this review. First, while the respective checklist assessed each study for quality, our process was still subjective. There is a possibility that some articles were overlooked, which could have accentuated this review. Likewise, articles from only the last five years were screened to collate the information presented. There may have been information from older articles that may have underscored an idea or filled the information gap. Moreover, most of the studies only had samples of cancerous breast tissue available for analysis. The studies were not done on actual patients, and therefore, side effects and overall quality of life were not assessed. The actual efficacy and adverse effects of the compounds mentioned are yet to be determined.

## Conclusions

The purpose of the study was to summarize the literature concerned with the SphK/S1P axis in breast cancer. We looked at the physiology of sphingolipid metabolism before describing what occurs in breast cancer cells. The focus then shifted to the effects the tumor had on the TME due to its importance in tumor survival. Lastly, we analyzed the compounds acting on SphK1 and SphK2.

While summarizing the SphK/S1P axis, we found that more research is needed. In our opinion, areas that need to be emphasized include identifying and solidifying the exact role of the SphK/S1P axis at the every level from genetic and intracellular expression to the TME. Specifically, more studies are needed to understand lymphangiogenesis in regards to SphK. Likewise, testing the discovered compounds and drugs at the next stage is also essential. It was shown that SphK inhibitors could have a synergistic action with other drugs on the market. Clinical trials are needed to dissect these compounds thoroughly. On the same note, nanoparticles carrying a combination of therapy for breast cancer is novel and needs further evaluation. By compiling the information thus far, we hope that our effort is a stepping stone for eventual clinical research.
